# Inference of gene regulatory networks from time series by Tsallis entropy

**DOI:** 10.1186/1752-0509-5-61

**Published:** 2011-05-05

**Authors:** Fabrício Martins Lopes, Evaldo A de Oliveira, Roberto M Cesar

**Affiliations:** 1 Federal University of Technology - Paraná, Brazil; 2 Institute of Mathematics and Statistics, University of São Paulo, Brazil; 3 Brazilian Bioethanol Science and Technology Laboratory (CTBE), Brazil

## Abstract

**Background:**

The inference of gene regulatory networks (GRNs) from large-scale expression profiles is one of the most challenging problems of Systems Biology nowadays. Many techniques and models have been proposed for this task. However, it is not generally possible to recover the original topology with great accuracy, mainly due to the short time series data in face of the high complexity of the networks and the intrinsic noise of the expression measurements. In order to improve the accuracy of GRNs inference methods based on entropy (mutual information), a new criterion function is here proposed.

**Results:**

In this paper we introduce the use of generalized entropy proposed by Tsallis, for the inference of GRNs from time series expression profiles. The inference process is based on a feature selection approach and the conditional entropy is applied as criterion function. In order to assess the proposed methodology, the algorithm is applied to recover the network topology from temporal expressions generated by an artificial gene network (AGN) model as well as from the DREAM challenge. The adopted AGN is based on theoretical models of complex networks and its gene transference function is obtained from random drawing on the set of possible Boolean functions, thus creating its dynamics. On the other hand, DREAM time series data presents variation of network size and its topologies are based on real networks. The dynamics are generated by continuous differential equations with noise and perturbation. By adopting both data sources, it is possible to estimate the average quality of the inference with respect to different network topologies, transfer functions and network sizes.

**Conclusions:**

A remarkable improvement of accuracy was observed in the experimental results by reducing the number of false connections in the inferred topology by the non-Shannon entropy. The obtained best free parameter of the Tsallis entropy was on average in the range 2.5 ≤ *q *≤ 3.5 (hence, subextensive entropy), which opens new perspectives for GRNs inference methods based on information theory and for investigation of the nonextensivity of such networks. The inference algorithm and criterion function proposed here were implemented and included in the DimReduction software, which is freely available at http://sourceforge.net/projects/dimreduction and http://code.google.com/p/dimreduction/.

## 1 Background

In general, living organisms can be viewed as net-works of molecules connected by chemical reactions. More specifically, the cell control involves the activity of several related genes through gene networks, with the relationship among them being generally broadly unknown. The inference or reverse-engineering of such gene networks is very important to uncover the functional relationship among genes and can be defined as the identification of gene interactions from experimental data through computational analysis [1].

Gene regulatory networks (GRNs) are used to indicate the interrelation among genes in the genomic level [2]. Such information is very important for disease treatment design, drugs creation purposes and to understand the activity of living organisms in the molecular level. In fact, there is a strong motivation for the inference of GRNs from gene expression patterns, *e.g.*, motivating the DREAM project [3].

The development of techniques for sampling expression levels of genes along time has increased the possibility of important advances in the understanding of regulatory mechanisms of gene transcription and protein synthesis. In this context, an important task is the study and identification of high-level properties of gene networks and their interactions, without the necessity of low-level biochemical descriptions. It is not the objective of this work to analyze a detailed biochemical model. The objective is to recover the gene connections in a global and simple way, by identifying the most significant connections (relationships).

While it is not possible to infer the network topology with great accuracy using only gene expression measurements mainly due to the short sample sets and the high system dimension, *i.e.*, the number of genes, as well as its complexity [4], the use of such inferences can be very important for planning experiments and/or to focus in some small meaningful subgroups of genes, thus reducing the complexity of the problem.

We are interested in the inference of network topology from temporal expression profiles by minimizing the conditional entropy between the genes, *i.e.*, the gene entropy conditioned to the state of others genes. Given a gene, the idea is to set as predictors the genes that minimize its entropy. Therefore, the conditional entropy works as a criterion function which has to be minimized. As in a typical machine learning problem, the quality of the inference depends on the data and the criterion function. If the data is not representative, the obtained solution will probably not be a global minimum but a local one. Similarly, if the criterion function is not suitable, the solution can only partially satisfy the constraint imposed by the data or even represent a wrong solution. Of course, since the criterion function follows the properties of the entropy concept, a completely wrong solution is not expected. In other words, if the observation of some genes reduces the uncertainty on the target gene, the prediction accuracy is improved. But it may not be the best or optimal one, which brings the question: *what is the best entropy function for the inference of GRNs?*

In this paper we address this question by presenting a new criterion function for the inference of GRNs in order to introduce the sensibility of the minimum conditional entropy regarding its functional form. The generalized entropy functional form proposed by Tsallis [5] is adopted, which not only recovers the Shannon form but also presents properties required by the *Statistical Physics Theory*. These properties are related to Thermodynamics principles, to the concept of stability and its axiomatic foundations [6].

A variety of statistical methods to infer network topology has been applied to gene expression data [1,7-20]. The results are often evaluated by comparing predicted couplings with those known from biological databases. While this procedure can elucidate or corroborate inferred interactions between some couples of genes, it has the drawback of the difficulty in estimating the false detection rate [4] and thus making the validation process very difficult. As it is not always possible to assure the quality of inference methods by analytical calculus, mainly in high complex systems, it is very important to use computational experiments to do it. Besides, in such experiments (simulations) it is possible to investigate prior information, as topology classes (*e.g.*, random or scale-free networks), or the system dynamics. Therefore, an *Artificial Gene Network (AGN) *platform [21,22] and the DREAM4 *in silico *network challenge [3] are explored in the present paper in order to assess the GRNs inference process by generalized entropy introduced in the present paper.

## 2 Results and Discussion

### 2.1 Experiments

In order to verify the effect of the entropic parameter *q*, we carried out inference experiments considering two types of network topologies: the uniformly-random Erdös-Rényi (ER) and the scale-free Barabási-Albert (BA) models [23-25]. In the ER model each connection (edge) is present with equal probability, in such way that the probability distribution of the connectivity of the genes follows a Binomial or Poisson distribution, with mean = 〈*k*〉. On the other hand, in the BA model the probability of a new node *v_j _*be connected to the node *v_i _*is proportional to the connectivity of *v_i_*, which produces a power-law in the probability distribution of the connectivity.

The data set *D_T _*was generated according to Sec. 4.3.2 with *N *= 100 (the number of genes). For each type of network model 10 sequences of 30 transitions starting from random initial states were generated, which are obtained by applying Boolean transition functions. Then, the 10 segments were concatenated into a single expression profile, which was submitted to the network inference method. The inference was made by means of Equation 6 with *q *varying from 0.1 to 3.1 in steps of 0.1 and from 3.1 to 10.1 in steps of 0.5, *i.e.*, the similarity between the source and the inferred AGN was calculated to each *q *in this range.

**Figure 1:**
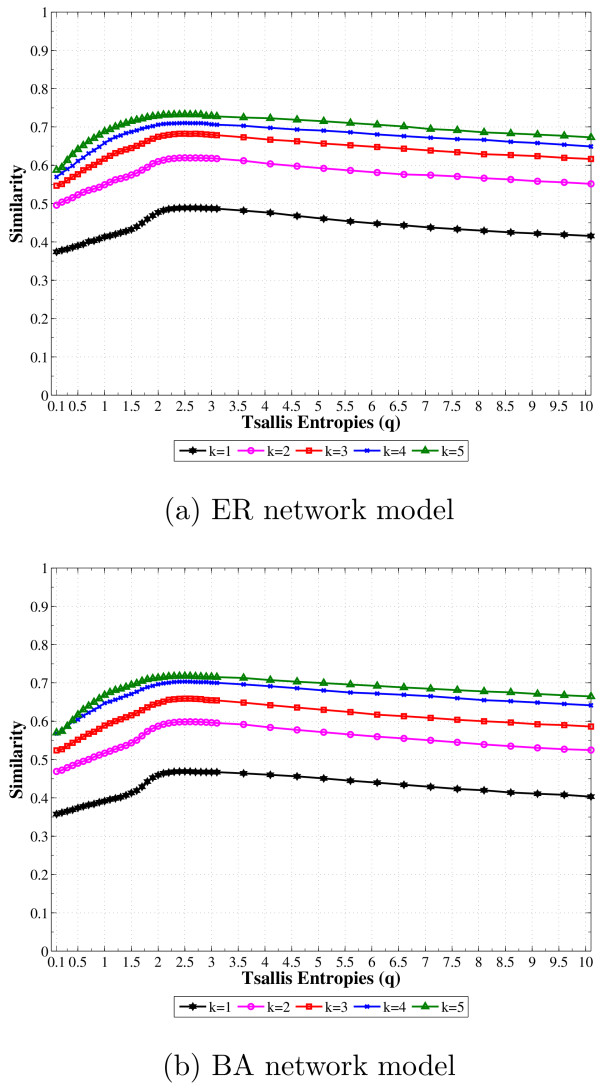
**Similarity between AGNs and inferred networks as a function of the entropic parameter *q***. Similarity between source and inferred networks as a function of the entropic parameter *q *for each average connectivity (1 ≤ 〈*k*〉 ≤ 5): (a) average values for the uniformly-random Erdös-Rényi (ER) and (b) average values for the scale-free Barabási-Albert (BA). The simulations were performed for 100 genes (N = 100) and represent the average over 50 runs.

**Figure 2:**
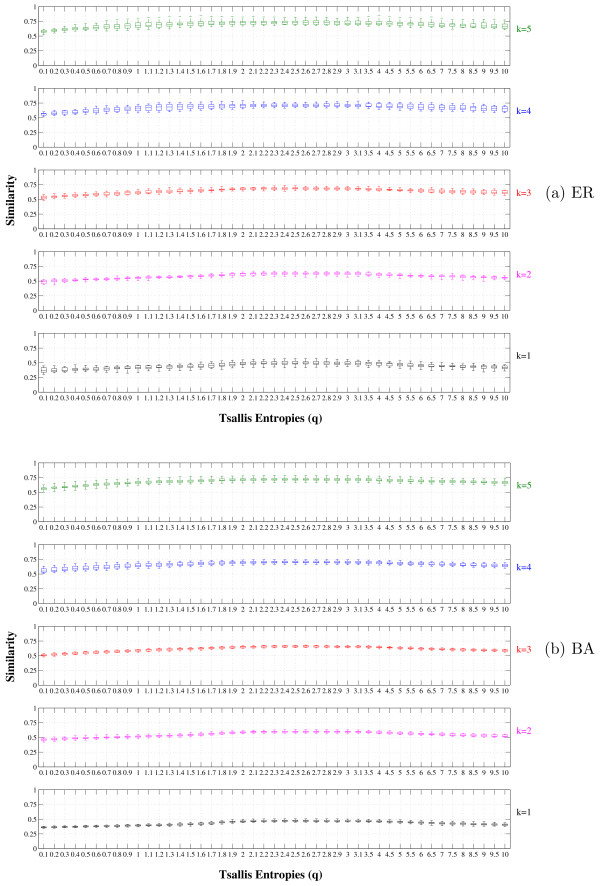
**Distribution of the similarity values between AGNs and inferred networks as a function of the entropic parameter *q***. Distribution of the similarity values between source and inferred networks as a function of the entropic parameter *q *for each average connectivity (1 ≤ 〈*k*〉 ≤ 5): (a) boxplot for the uniformly-random Erdös-Rényi (ER) and (b) boxplot for the scale-free Barabási-Albert (BA). The simulations were performed for 100 genes (N = 100) and represent the average over 50 runs.

The similarity curves shown in Figure 1 were obtained by averaging 50 runs (different source networks) for each network model. In both network models improvements were observed in the similarity by ranging *q*, with the maximum 〈*Similarity(A, B)*〉 being reached by *q *≠ 1 for all tried 〈*k*〉. Besides, it can also be noted that the q* that maximizes the similarity seems to be almost independent of the network model and the average connectivity. Figures 2(a) and 2(b) show the boxplots of the similarity values for each *q *and *k *values. It is possible to notice a very small variation in the boxplots, indicating stable results for all *q *values.

**Figure 3:**
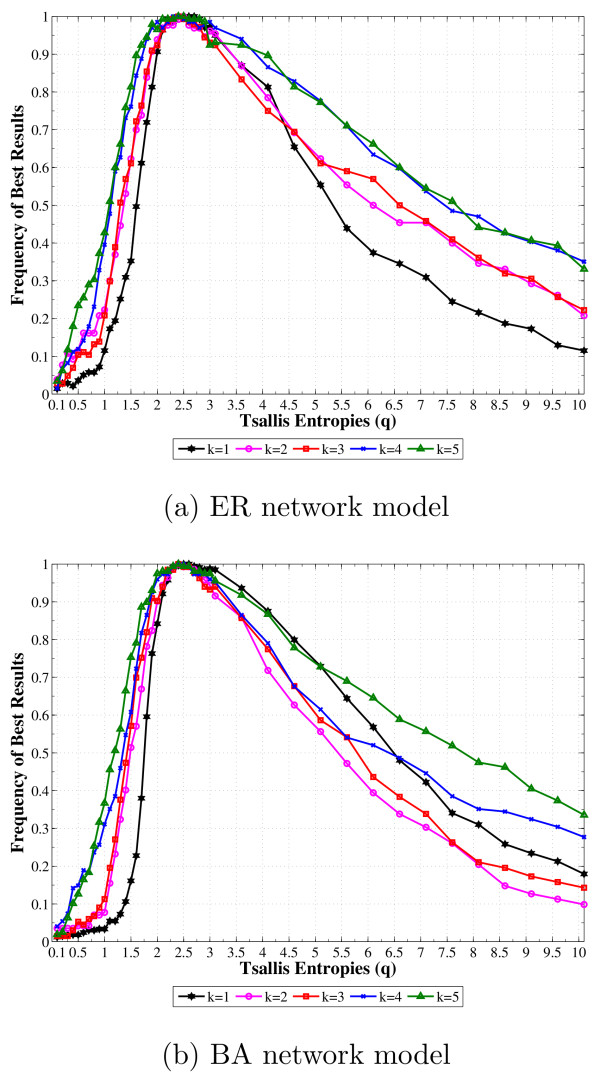
**Frequencies where the *q *value appears in the better answer for AGNs**. Frequencies where the *q *value appears in the better answer for each target gene and for each average connectivity (1 ≤ 〈*k*〉 ≤ 5): (a) uniformly-random Erdös-Rényi (ER) and (b) scale-free Barabási-Albert (BA). The simulations were performed for 100 genes (N = 100) and represent the average over 50 runs.

In order to better investigate this behavior, Figure 3 shows the normalized frequency curves of the best *q *for each gene in the sense of higher similarity. It is clearly observed that higher frequencies are concentrated in the range 2 ≤ *q *≤ 3 for both network models and varied connectivity. This indicates and reinforces (Figure 1) a non-dependence on the topology network in the improvement of the inference by taking non-Shannon entropy (*q *≠ 1).

In particular, considering the frequency curves in Figure 3, the average q* was calculated for each network model given the average connectivity. These averages seem to be almost constant (around 3.20 for the ER model and 3.23 for the BA model) as well as the *q*'s with higher frequencies, *i.e.*, maximum amplitude in the frequency curves.

In order to confirm our findings, we also evaluate the behavior of the proposed methodology by using the DREAM4 *in silico *network challenge [3]. In this challenge the time series data was considered, which provides five different networks of size 10 and other five of size 100. The networks of size 10 have 5 different time series, while the networks of size 100 have 10 time series. Each time series has 21 time points generated from a differential equations model with noise. The DREAM4 *in silico *network challenge has 5 and 10 time series with 21 time points each, which were also concatenated to form a single expression profile, similarly to the previous case (AGNs).

**Figure 4:**
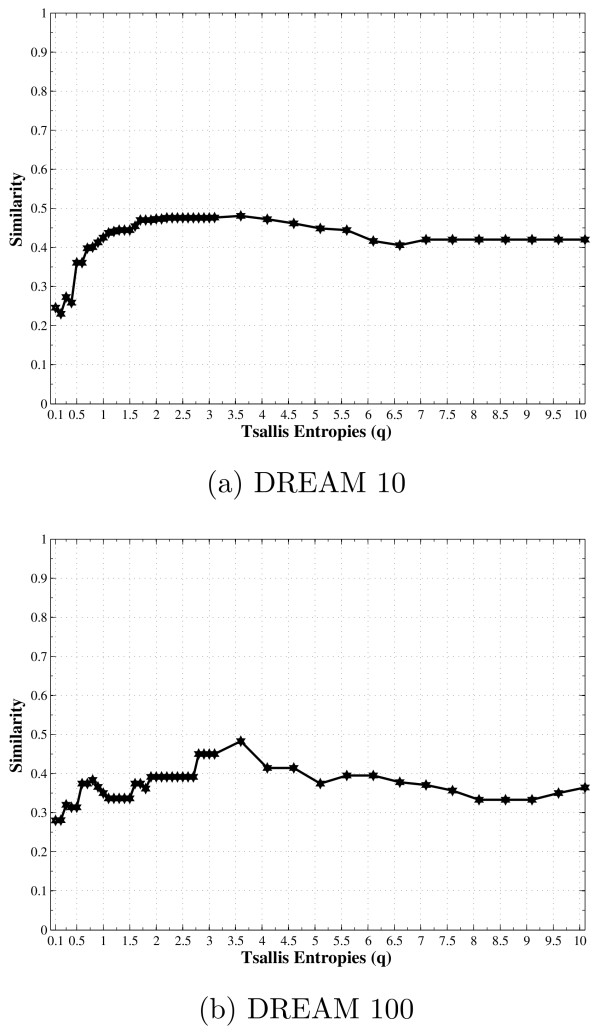
**Similarity between DREAM and inferred networks as a function of the entropic parameter *q***. Similarity between gold standard and inferred networks as a function of the entropic parameter *q*, by considering the average results over the five DREAM networks available of each size: 10 and 100 genes.

The same methodology was applied with the similar used parameters. Only one additional step was included for the quantization of the DREAM data. The proposed criterion function and the adopted methodology are based on entropy calculations, in which a step of data quantization may be required if the original input data is not discrete, is the case of DREAM data. The applied method for the quantization process is described in [26]. It was applied by considering 2 levels for networks of size 10 and 3 levels for networks of size 100. In this context, an integer value represents each quantization level used by the quantization process. For example, 2 levels means that the quantized signal has only 0's and 1's. Then, each quantized network signal was submitted to the same methodology adopted in the present pa-per. Figure 4 presents the average results obtained for each DREAM network size: 10 and 100. It is possible to notice an improvement on the similarities by varying the parameter *q*, in which the best results were obtained by *q *≠ 1 for the two network sizes.

**Figure 5:**
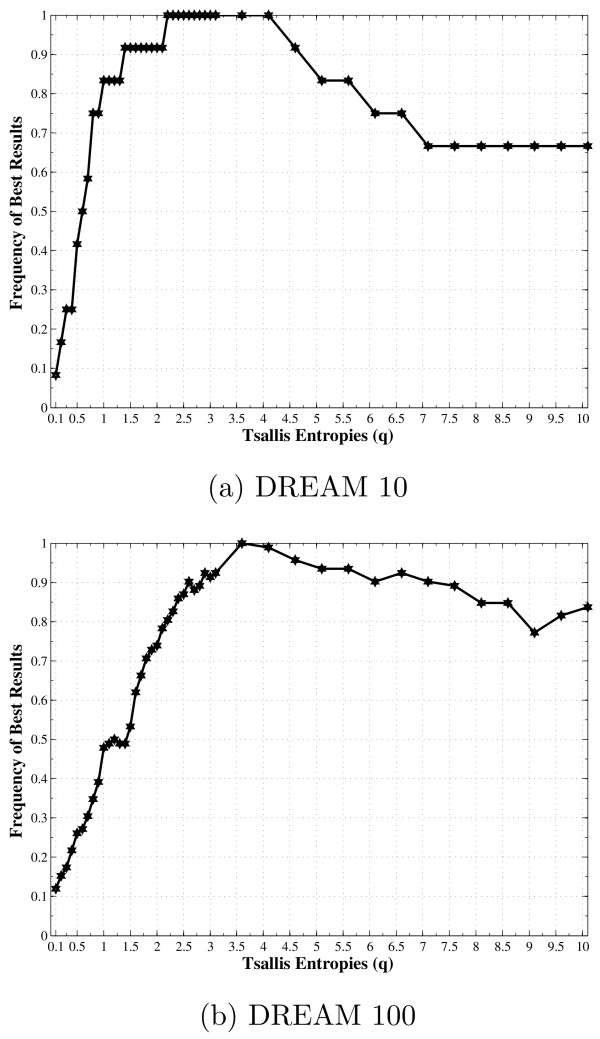
**Frequencies where the *q *value appears in the better answer for DREAM networks**. Frequencies where the *q *value appears in the better answer for each target gene, by considering the average results over the five DREAM networks available of each size: 10 and 100 genes.

Figure 5 presents the normalized frequency, in which the *q *value was able to infer the best set of predictors (higher similarity) for each gene. The higher frequencies are concentrated in the range 2.2 ≤ *q *≤ 4.1 for the DREAM network of size 10 and 3.2 ≤ *q *≤ 5.5 for the DREAM network of size 100. Regarding the frequency curve in Figure 5, the average q* was calculated for each network size, being around 3.30 for the DREAM 10 and 3.92 for the DREAM 100, which are similar to those presented for ER and BA networks, but with slightly higher value for DREAM 100 network. It is important to highlight the existence of a range of *q *values that produce better results, on average 2.5 ≤ *q *≤ 3.5 (subextensive entropy).

All experimental results confirm that the proposed criterion function can improve the accuracy of the inference process, thus indicating that the network nonextensivity is an important matter of investigation for inference methods based on information theory. As a result, it achieved a better accuracy on the inference of GRNs from gene expression patterns.

## 2.2 Discussion

The use of the entropy or mutual information as a criterion function on the problem of network inference is not new and has been largely applied for the inference of GRNs in recent years [1,10,11,13,14,16,17,19,20]. This is explained by the possibility that some genes may be well predicted by observing states of other genes in a regulatory network, which makes the use of conditional entropies suitable. If the relationship between these genes are linear, a simple Pearson correlation analysis would be enough to get a good description of the gene network. However, when the relationship between genes is not linear but it is described by functions of more than one predictor gene, it is expected that the inference by methods based on the entropy concept produces better results than those based on Pearson correlation. Naturally, this leads to the necessity of investigating the sensibility or robustness of these methods with respect to the extensivity of the applied entropy. In this context, it was verified in a previous work [27] that the entropic parameter *q *was very important to achieve better results in the GRNs inference process. In the present work, we introduce a criterion function by adopting the generalized Tsallis entropy in order to verify the dependence of the inference on the entropy functional form and characterize how this dependence occurs.

The experimental results provide more evidence about the sensibility of the inference process to the extensive/nonextensive entropies. In addition, the experimental results indicate that the nonextensivity property of the entropy is an important factor to be investigated and explored in the GRNs inference process in order to improve its accuracy, thus opening new perspectives for inference methods based on the entropy minimization principle.

As expected, we observed different similarity scores for different entropic parameters *q*. The maximum similarity score for all tried network models was reached by *q *≠ 1, with an improvement of 20% compared to the similarity score for *q *= 1 (see Figure 1 and 4). In order to better visualize the relevance of this improvement, it is important to take a look closer on the correctly and incorrectly inferred edges. For a network with *N *genes, *N*^2 ^directed edges are possible when every node is connected to itself and to each other, (*C_ij _*= 1 for all 1 ≤ *i*, *j *≤ *N *). As the simulations were made with 1 ≤ 〈*k*〉 ≤ 5, *C *was always a sparse matrix with the number of connections between the genes given by *T P *+ *F N *.

Table 1 presents the best number of correctly and incorrectly inferred edges by considering each gene individually. It is possible to observe a very good accuracy of recovering correct edges (*T P *and *F P *) in the ER and BA model by adopting *q *= 2.5 (subextensive entropy). In this context, the recovery of false connections (FP) seems to be dependent of the best entropy functional form. On the other hand, the network model does not seem to be dependent. Therefore, in order to improve the inference it is necessary to introduce information about the class model in the method. Furthermore, another observed property that does not depend on the network class model is the reduction on the number of inferred false connections (FP), *i.e.*, when the algorithm infers a connection that does not exist between a pair of genes. This indicates a more conservative inference when an adjusted *q *is used, even for networks with high connectivity -- the number of FP connections for 〈*k*〉 = 5 obtained by the Shannon entropy was more than six times greater than that obtained by the generalized entropy with the adjusted *q *= 2.5 for the BA networks and more than eight times greater for the ER networks.

**Table 1:** The best results found for *q *= 2.5 compared with *q *= 1.0.

(a) ER network model					
***q***	***k***	**TP**	**FP**	**FN**	**TN**

1	1	223	229	21	9735
2.5		229	105	15	9741

1	2	311	162	27	9635
2.5		320	50	18	9644

1	3	344	131	36	9584
2.5		362	17	18	9602

1	4	381	96	46	9527
2.5		397	15	30	9543

1	5	383	96	91	9435
2.5		401	11	73	9453

					
**(b) BA network model**					

***q***	***k***	**TP**	**FP**	**FN**	**TN**

1	1	168	285	47	9738
2.5		175	149	40	9745

1	2	251	212	30	9689
2.5		259	83	22	9697

1	3	304	156	55	9586
2.5		314	37	45	9596

1	4	348	125	102	9448
2.5		356	21	94	9456

1	5	360	110	117	9406
2.5		383	16	94	9429

The best results found for *q *= 2.5 compared with *q *= 1.0 by considering each gene individually in the same network: (a) uniformly-random Erdös-Rényi model (ER) and (b) scale-free Barabási-Albert network topology (BA).

It was also observed that distributions with mass concentrated in one of the classes are less penalized by applying *q *values near to 2.5. By considering that the system (organism) has a stochastic behavior and can receive external perturbations, it is expected that the class distributions are not deterministic among the possible classes, *i.e*, in binary case 0 or 1. In other words, given the nature of the system it is desirable from method to infer connections from classes with concentrated distributions and few errors among its classes (Table 2(b)) compared to more uniform distributions in one of the classes and no errors in the other (Table 2(a)). An important observed issue is that subextensive entropies, *e.g.*, *q *values near to 2.5, promote this preference in the presented inference method. Table 2 shows an example of probability distribution that illustrates this situation. The predictor states are on the first column and the number of observed states for the target states on columns two and three, thus generating a mass probability distribution table for a target gene by observing its predictor states. In columns four, five and six we have the criterion function results (conditional entropy) for each distribution by using different *q *values. The mean conditional entropy results marked with * represent the minimal achieved by the method, and therefore selected as predictor for the target by the inference method.

**Table 2:** Example of change on the inferred predictor by using different values for *q *entropic parameter.

(a)					
	**Target**		**Criterion Function Results**		

**Predictor A**	**0**	**1**	**q = 0.5**	**q = 1**	**q = 2.5**

0	18	23	0.108	0.090	0.056
1	278	0	0	0	0

**mean conditional entropy **			0.108*	0.090*	0.056

					
**(b)**					

	**Target**		**Criterion Function Results**		

**Predictor B**	**0**	**1**	**q = 0.5**	**q = 1**	**q = 2.5**

0	1	16	0.024	0.013	0.005
1	295	7	0.265	0.104	0.036

**mean conditional entropy**			0.289	0.117	0.041*

Example of change on the inferred predictor by using different values for *q *entropic parameter: (a) distributions that lead to wrong predictor and (b) distributions that lead to correct predictor.

**Figure 6:**
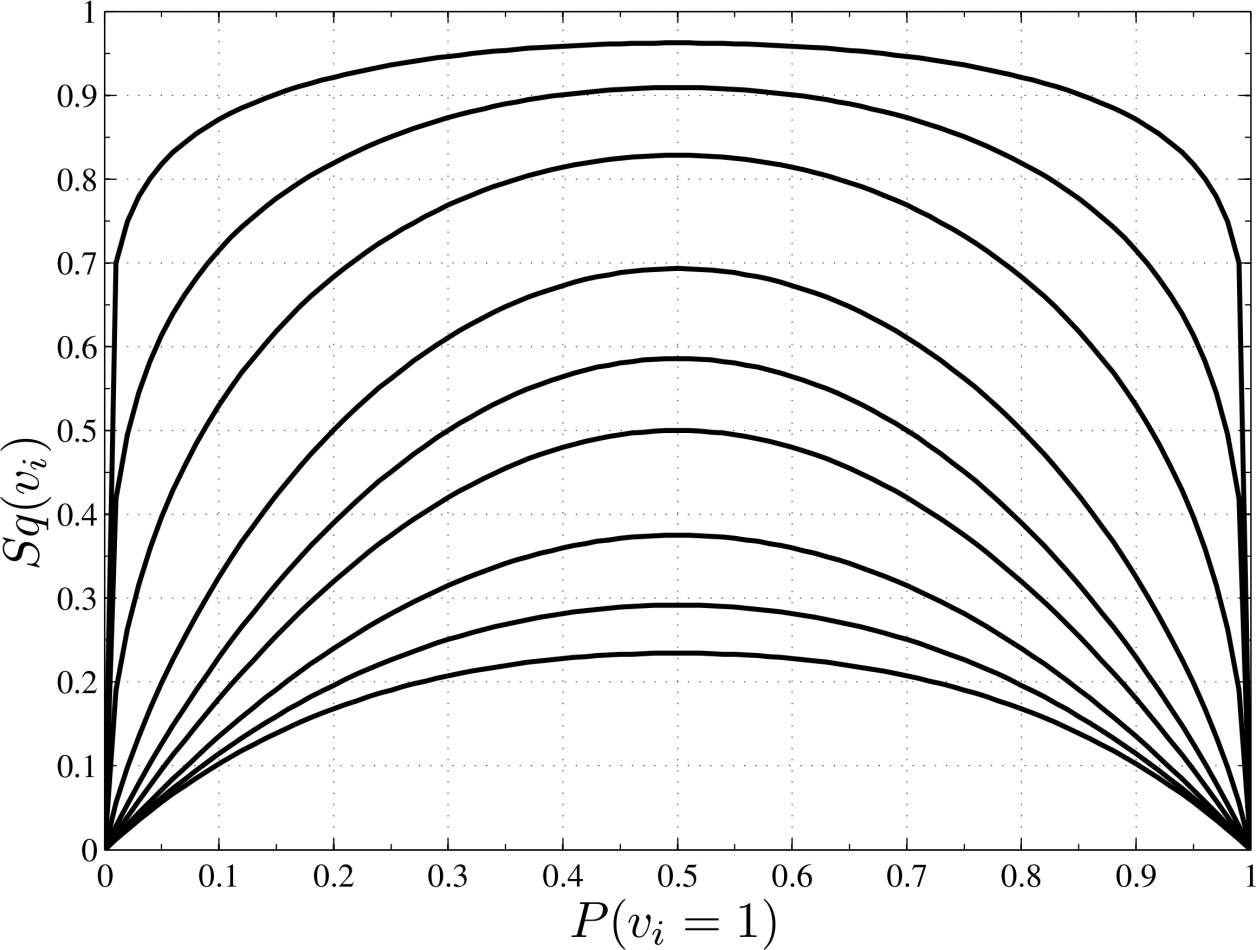
**Generalized entropy *S_q _*as a function of the probability *P*(*v_i _*= 1)**. Generalized entropy *S_q _*as a function of the probability *P*(*v_i _*= 1) for binary genes, *v_i _*∈ {0, 1}. From up to bottom, the curves were obtained with *q *set to: 0.1, 0.25, 0.5, 1.0, 1.5, 2.0, 3.0, 4.0, 5.0.

As we can see, the minimum criterion function score changes with *q *and so the gene will be selected as predictor. For *q *= 0.5 and 1.0 the method selected gene *A *as best predictor, while gene *B *is selected for *q *= 2.5. For almost probable states, the derivative of the generalized entropy increases as *q *decreases (see Figure 6). This behavior allows *S_q _*(*target*|*B *= 1) to be significantly greater than *S_q _*(*target*|*A *= 1) depending on *q*. In this context, distributions concentrated in one of the classes (few errors) can produce higher conditional entropy values, which can be very amplified by the predictor distribution mass. Therefore, when *q *= 0.5 or 1.0 the method selects the predictor gene *A *since it induces a null entropy on the target (when *A *is active), besides the high entropy on the target induced when it (gene *A*) is inactive. However, when *q *is set to 2.5 (subextensive entropy) the balance between the conditional entropy and the predictor probability mass is adjusted in order to produce better accuracy on the inference process.

In summary, this situation characterizes how the subextensive entropy (*q *= 2.5) produces better results. In this example, it is considered a single target gene with a fixed number of time points on its expression data. Hence, Table 2(a) and 2(b) characterize two conditions of frequencies distribution that produce different predictors for the same target gene by using different values of *q*, in which *q *= 2.5 (subextensive entropy) achieves the correct predictor for the target. This example illustrates the trade-off between the conditional entropy of the target and the probability distribution of the predictor.

Tables 1(a) and 1(b) present the results obtained by a single value of the entropic parameter *q *= 2.5, in order to show how the improvements are achieved by fixing *q *value on the range 2.5 ≤ *q *≤ 3.5 (subextensive entropy). However, the main point in the Tsallis Theory is that there is not an universal *q *that should be used on every data set. The optimal *q *should be set by the system (or kind of systems), *e.g.*, we have observed that for Boolean networks this value was found close to 2.5 and 3.5 for the DREAM networks. If we pay attention to the Figures 2(a) and 2(b), it will be noted that not only the averaged similarity is improved by considering *q *= 2.5 instead of *q *= 1, but also the best and worst inferences (the highest and lower line in the boxplot) obtained in the sample dataset. Besides, it can also be observed the variance in the similarity is almost constant with respect to *q *(*q *= 1 and *q *= 2.5) for low levels of connectivity (small *k*) and reduced for high levels of connectivity (large *k*) when *q *= 2.5.

An important property of the GRNs inference method regards stability. The boxplots results shown in Figures 2(a) and 2(b) present very small variations for all tested *q *values. These results are an important indicative of the stability of the proposed methodology.

## 3 Conclusions

In general, reverse-engineering algorithms using time series data need to be improved [1]. The present work opens new perspectives for methods based on information theory, in face of all results discussed which show a relevant improvement on the inference accuracy by adopting nonextensive entropies proposed by Tsallis. In particular, the subextensive entropies provide a remarkable improvement of accuracy by reducing the number of false connections detected by the method. The obtained experimental results showed the importance of the range of values 2.5 ≤ *q *≤ 3.5 (subextensive entropy).

An interesting point regards the logic circuits created by Boolean functions and its dynamics. The inference method finds some of them independent of the *q *value, while others are found by tuning this parameter, as presented in the previous section. Future works should investigate the Boolean functions or logic circuits that are sensitive to entropic parameter *q *and the local structures formed by them.

The inference algorithm and criterion function described in this work were implemented and included in the DimReduction software [26], which is freely available at http://sourceforge.net/projects/dimreduction and http://code.google.com/p/dimreduction/.

## 4 Methods

### 4.1 Selecting predictors by conditional entropy

The mutual information may be understood as a measure of the dependence between variables, with this dependence being quantified by calculating the average amount in the uncertainty on some variable *v_i _*given the knowledge about other accessible variable *v_k_*, and vice-versa. In this sense, the mutual information indicates how much the prediction error of the state of *v_i _*changes if we know the state of *v_k_*.

Given two random variables *v_i _*and *v_k_*, their mutual information can be calculated by [28]:(1)

where

are the Boltzmann-Gibbs entropy of the gene *i *and its conditional entropy on the gene *v_k_*, also known as the Shannon entropy and its conditional entropy, respectively.

If the states of the genes taken into account in Equation 1 are collected in distinct times, *i.e.*, *v_i_*(*t*+1) and *v_k_*(*t*), the mutual information can be used to select predictor genes (*v_k_*(*t*)) as those that minimize the uncertainty on the target gene (*v_i_*(*t *+ 1)). Thus, the method consists in finding the gene *v_k _*that maximizes Equation 1 for a given target gene *v_i_*, which is equivalent to find the gene *v_k _*that minimizes the conditional entropy *S*(*v_i_*(*t *+ 1)|*v_k_*(*t*)). Despite the symmetry in *I*(*v_i_*(*t *+1), *v_k_*(*t*)) with respect to the variables *v_i_*(*t *+ 1) and *v_k_*(*t*), since the state variables computed in it belong to different time instants, *t *and *t *+ 1, it is possible to infer a causality between *v_i_*(*t *+ 1) and *v_k_*(*t*). As *I*(*v_i_*(*t *+ 1), *v_k_*(*t*)) is not necessarily equal to *I*(*v_k_*(*t *+ 1), *v_i_*(*t*)), this causality can be estimated by the difference between *I*(*v_i_*(*t*+1), *v_k_*(*t*)) and *I*(*v_k_*(*t*+1), *v_i_*(*t*)) or, in a simple way, by *S*(*v_i_*(*t *+ 1)|*v_k_*(*t*)).

Naturally, the mutual information is not restricted to pairs of genes and we can use it to infer the dependence of *v_i _*on groups of genes: *I*(*v_i_*(*t *+ 1); {*v_j _*...*v_k _*}(*t*)) = *S*(*v_i_*(*t *+ 1)) - *S*(*v_i_*(*t *+ 1)|{*v_j _*...*v_k _*}(*t*)). Therefore, given a set *D *of temporal gene expression profiles from a network, the method looks for the group of genes that maximizes Equation 1 for each gene. If *I*(*v_i_*(*t *+ 1); {*v_j _*...*v_k_*}(*t*)) presents the maximum score calculated from *D*, then each gene of {*v_j _*...*v_k_*} is directly connected to *v_i _*as predictor. In the same way, if there is not a group that causes significantly variations on the mutual information, then *v_i _*is selected as a source or an isolated gene (in the case that *v_i _*is not selected as a predictor of any gene). Once the method is applied to each gene individually, the individual entropy of the target *v_i _*(*S*(*v_i_*(*t *+ 1))) is kept constant during the search for predictors, and as a result the method returns as predictors the genes that produce the lowest conditional entropy (*S*(*v_i_*(*t *+1)|{*v_j _*...*v_k _*}(*t*))). In other words, the mutual information can be calculated by the difference between the individual entropy *S*(*v_i_*(*t *+ 1)) and the mean conditional entropy *S*(*v_i_*(*t *+ 1)|{*v_j _*...*v_k_*}(*t*)), by considering a group of genes *g*(*t*) = {*v_j _*...*v_k_*}(*t*). Therefore, the difference between *I*(*v_i_*, *v_k_*) and *I*(*v_i_*, *g*) is due to the mean conditional entropy, once the individual entropy of *v_i_*, *S*(*v_i_*), is exactly the same in both *I*(*v_i_*, *v_k_*) and *I*(*v_i_*, *g*).

### 4.2 Beyond the Boltzmann entropy

The concept of entropy was introduced by Clausius in the context of Thermodynamics considering only macroscopic statements [29]. Motivated by the idea of relating it to the Classical Mechanics some years later, Boltzmann showed that this entropy could be expressed in terms of the probabilities associated to the microscopic configuration of the system [30]. However, in his mathematical demonstration there were some considerations about the nature of the physical system to assure the recovery of the properties of Clausius macroscopic entropy by his microscopic approach -- *e.g.*, short-range interactions, a necessary condition to assure the extensivity of the Boltzmann entropy [6,31]. Thus, despite the great importance and success of the Boltzmann entropy, there are situations were such conditions are not preserved [32] and Boltzmann entropy will hardly recover the properties of the Clausius entropy.

Inspired by the probabilistic description of multifractal geometry, C. Tsallis proposed in 1988 a generalization of the Boltzmann entropy [5] which, along two decades, has been successful in presenting desired properties of Statistical Physics Theory [6,33] with great experimental accordance [31].

The proposed definition is [5](2)

where *k *is a positive constant (which sets the dimension and scale), *w *is the number of distinct configurations of the system, *p_i _*is the probability of such configuration and *q *∈ ℛ is the *entropic parameter*.

The entropic parameter characterizes the degree of nonextensivity, which in the limit *q *→ 1 recovers  with *k *being set to the Boltzmann constant *k_B_*.

The Boltzmann-Gibbs entropy is said to be extensive in the sense that, for a system consisting of *N *independent but equivalent subsystems *v *= {*v*_1_*, v*_2_*, ..., v_N_*}, the entropy of the system is given by the sum of the entropy of the subsystems: *S*(*v*) = *NS*(*v*_1_) [31]. In the Tsallis entropy, this extensivity is set by the parameter *q*, which can be clearly visualized by the compound rule [31]:(3)

with A and B being independent systems, *i.e.*, *P*(*A,B*) = *P*(*A*)*P*(*B*). We can observe superextensivity for *q <*1, extensivity for *q *= 1 and subextensivity for *q >*1. More specifically, *S_q _*is always nonnegative for *q >*0. Although it is also possible to have *S_q _>*0 for some *q <*0, *q >*0 is generally used to avoid divergences or some inconsistencies [31].

Equation 2 has been largely applied to different physical problems, *e.g.*, http://www.cbpf.br/GrupPesq/StatisticalPhys/biblio.htm for a large bibliography, leading to good agreements with experimental data. Naturally, despite these applications, it can be asked if the Tsallis entropy is also suitable to code information in a general way such as Shannon [34], Khinchin [35] and Kullback [36] showed to be the Boltzmann entropy. Some papers have been published verifying the mathematical foundation of the Tsallis entropy, similarly to the axiomatic approach used by Khinchin [37,38], as well as investigating its nonaddictive features and their interpretations [6,39]. As in typical physical problems, there are some examples where the Boltzmann-Shannon entropy is not suitable [40]. Besides, it is also possible to define a divergence equivalent to the Kullback-Leibler [41].

By defining ln*_q_*(*x*) ≡ (*x*^1-*q *^-1)*/*(1 - *q*), Equation 2 can be written in a similar form of the Boltzmann entropy . In this way, a *generalized mutual information *between *v_i _*and *v_k _*can be defined as [41]:(4)

The generalized mutual information has the necessary properties to be used as a criterion measure for consistent testing [42] and, as Equation 1, it reaches its minimum value when *P*(*v_i_*|*v_k_*) = *P*(*v_i_*) and the maximum when  vanishes [41], which is equivalent to make  vanish. It is hence possible to look for dependencies between *v_i _*and *v_k _*by minimizing *S_q_*(*v_i_*|*v_k_*).

For binary genes, *v_i _*∈ {0, 1}, we have *S_q_*(*v_i_*) = [*P*(*v_i _*= 1)*^q ^*+ (1 - *P*(*v_i _*= 1))*^q ^*-1]*/*(1 - *q*) and the influence of the entropic parameter *q *can be easily observed. In Figure 6 the maximum entropy for the gene increases as *q *decreases, taking the limit  as *q *→ 0. Indeed, when *q *≈ 0, *S_q_*(*v_i_*) will be significantly different of  for *P*(*v_i _*= 1) ≈ 0 or *P*(*v_i _*= 1) ≈ 1, which means a very rigid criterion in the sense that, either the predictor candidates fulfill all the constraints imposed by the data or they can not be selected as predictors. On the other hand,  for *q *≫ 1 which can be interpreted as a very flexible criterion function in the sense that any gene or group of genes can be selected as good predictors.

Another interesting point is the ordering of the entropy with respect to *P*(*v_i _*= 1). If the entropy of *P*(*v_i _*= 1) = *a *is larger than the entropy of *P*(*v_i _*= 1) = *b *for a given q*, then it will always be large for any *q *-- see Figure 6. But this ordering is not preserved on the mean conditional entropy. For *S_q_*(*v_i_*|*v_k_*) the entropy of *v_i _*given *v_k _*is weighted by the probability of *v_k_*,(5)

in such way that it is possible to have  and  for some *q*' ≠ *q*″ and where the index *a *represents the constraint {*P*(*v_i _*= 1|*v_k _*= 0) = *a*_0_*, P*(*v_i _*= 1|*v_k _*= 1) = *a*_1_} and *b *the {*P*(*v_i _*= 1|*v_k _*= 0) = *b*_0_*, P*(*v_i _*= 1|*v_k _*= 1) = *b*_1_}. This results in a trade-off between the relevance of the conditional entropy and the probability distribution of the predictor genes.

In the context of feature selection or dependence variables test, in which the entropy is used as a criterion function, this non-preservation of the ordering means the existence of an optimal q* by which a system can be best reproduced. As in physical problems, q* should be related to the system properties [31] and discovering the laws or principles which relate q* to these properties becomes fundamental to improve recovering methods.

### 4.3 Proposed Method

The algorithm is based on previous works [8,11], which consists in looking for the group of genes that minimizes the criterion function (*i.e.*, conditional entropy) of the target gene. Therefore, for each given target *v_i _*we have to calculate the conditional probabilities *P*(*v_i_*(*t*+1)|*v_j_*(*t*), ..., *v_k_*(*t*)) based on the data set *D_T _*= {*s*(1), *s*(2), ..., *s*(*T *)}, where *s*(*t*) ≡ [*v*_1_(*t*), *v*_2_(*t*), ..., *v_N_*(*t*)] is the expression vector at time *t*, *i.e.*, the state of the network at time *t*.

For a network with *N *genes we have  conditional probabilities to be calculated for each gene, *i.e.*, *n_p _*possible groups of predictors. Fortunately, it is not expected that the genes are regulated for many predictors [43,44] and an upper bound for *n_p _*can be defined. Kauffman observed that chaotic dynamics are more probable for gene networks with *n_p _*≥ 3 [43,44] and by stability principles he concluded that the average connectivity should be upper bounded by three, once the gene network could be in the frontier of chaos but not chaotic. Herein, we relax a little the Kauffman statement and set this upper bound on the average connectivity 〈*k*〉 ≤ 5.

Another important point is the possibility of gene networks with different topology classes. In order to verify the sensibility of the method on the topology class, the topology of gene networks were generated with the uniformly-random Erdös-Rényi (ER) [45] and with scale-free Barabási-Albert (BA) [46] models. The BA complex network model is one of the most similar to known real regulatory networks [47,48]. Biological network topologies based on *Escherichia coli *and *Saccharomyces cerevisiae *[49] were also considered.

We describe below how the artificial gene networks were generated, the algorithm of inference, evaluation metrics and the experimental results.

#### 4.3.1 The inference algorithm and criterion function

Given the temporal data *D_T _*the algorithm fixes a gene target *v_i _*and looks for the group of genes *g *that minimizes the conditional entropy *S_q_*(*v_i_*(*t *+ 1)|*g*(*t*)) for a fixed *q*. As the network size is generally high, the search space becomes very high such that an exhaustive search is not appropriate. Then, we apply the *Sequential Forward Floating Search *(SFFS) [50] to circumvent this combinatorial explosion.

For the calculation of the conditional entropy (Equation 5) it is necessary to estimate the conditional probabilities of the target given its predictor candidates as well as the probabilities of these candidates. In the absence of prior information, these probabilities are estimated by the relative frequencies on *D_T_*, which means an accuracy dependence on the representativity of *D_T _*. Once we are searching for the lower entropy, it is not recommended to set the probability of non-observed instances as null. It is possible that some of the instances are not present in the temporal expression profile because of its small size sample and/or by the dynamics of the system, *i.e.*, the transition functions. Therefore, in order to reach a good trade-off we follow the penalization of non-observed instances [26,51]. The penalized criterion function by adopting the generalized Tsallis entropy is defined as follows:(6)

where *α *≥ 0 is the penalty weight, *m *is the number of possible instances of the gene group *g *(predictors), *n *is the number of observed instances, *d *is the total number of samples and *r_g _*is the number of each observed instance of *g*.

If *α *is set to zero, we do not have any penalization and *P*(*g*) is estimated by its relative frequency on *D_T_*, *i.e.*, by calculating the terms . When *n *= *m*, the penalization term, first term in Equation 6, is canceled and *P*(*g*) is now estimated by a modulated relative frequency of the predictors, by adding *α *to all instances of *g*, *i.e.*,

and finally when *n < m*, the parameter *α *is considered *m *- *n *times for non-observed instances (sum), and *n *times for observed instances. Thus, in Equation 6 a positive mass of probability is assigned to the non-observed instances of the gene group *g *in the expression data, which is parameterized by *α*.

Furthermore, the penalization of the non-observed instances is weighted by the entropy of the target gene, *i.e.*, *S_q_*(*v_i_*). This is important because of the possibility of having a good description about a gene when its uncertainty is small, *i.e.*, the observed instances of the genes are enough to describe the dynamics of a target gene with small entropy. In this paper we set *α *= 1.

The inference algorithm consists in calculating the mean conditional entropy by using Equation 6 and looking for a group of genes that minimizes it. This search is performed by the SFFS algorithm.

#### 4.3.2 Artificial gene networks

The adopted AGN model was built based on the random Boolean network (RBN) approach [43,44,52]. This model yields insights into the overall behavior of large gene networks, allows the analysis of large data sets in a global way and represents some fundamental characteristics of real GRNs [53-57]. In a RBN model, the state of each gene is a binary variable set by Boolean functions of other genes. The possibility to model GRNs as Boolean networks stems from the switch-like behavior that the cell exhibits during regulation of functional states [52,58]. In this context, the gene state is mapped from a continuous expression to a two-level expression (on/off).

More specifically, an artificial gene network (AGN) is defined by a set *V *= {*v*_1_, *v*_2_, ..., *v_N_*} of *N *genes (nodes), a *N *× *N *adjacency matrix *C *(with *C_ij _*∈ {0, 1}) and a set *F *= {*f*_1_, *f*_2_, ..., *f_N_*} of *N *transitions functions. In the Boolean approach, each *f_i _*is a logical circuit of the non-null elements of the *i^th ^*row of *C *that sets the state of the gene *v_i_*. Then, the network state at time *t *+ 1 is a N-dimensional vector *s*(*t *+ 1) = [*v*_1_(*t *+ 1), *v*_2_(*t *+ 1), ..., *v_N_*(*t *+ 1)] resulting from the application of these functions to the previous state *s*(*t*). Besides, the connectivity of *v_i _*is given by  and the topology class of the network is defined by the probability distribution of these connectivities.

The networks used in this paper were obtained by the network generator proposed in [21,22]:

1. define a topology class, *i.e.*, the distribution *P*(*k*) of the number *k *of connections per gene;

2. define the *k_i _*connectivity for each gene *v_i _*setting the predictors (*C_ji_*'s) and targets (*C_ij _*'s) by using the *P*(*k*) distribution;

3. set the transfer function *f_i _*for each gene *v_i _*by random drawing a truth table according to its number of predictors , *i.e.*, an output state for each one of the  input states.

Once defined the AGN, the simulated temporal expression profile *D_T _*is obtained by defining an arbitrary initial state of the network and successive applications of the transfer functions.

On the other hand, DREAM4 temporal expression profiles were generated by considering network structures based on *Escherichia coli *and *Saccha-romyces cerevisiae *[49]. The dynamics was generated by continuous differential equations with the inclusion of perturbations on the data in order to simulate a physical or chemical intervention. Gaussian noise was also added in order to simulate expression measurement error. In summary, the DREAM4 time series database presents variations of network size with 10 and 100 genes, perturbation and noise on expression profiles generated by differential equations. A detailed description is provided in the DREAM project website [3].

In both cases (AGN and DREAM network), the temporal expression profile *D_T _*is submitted to the inference method and its results are evaluated according to the measures presented in the next section.

#### 4.3.3 Evaluation

In order to quantify the similarity between the source gene network *A *and the inferred network *B*, we adopted the validation metric based on a confusion matrix [59] (see Table 3).

**Table 3:** Confusion matrix.

Edge/Connection	Inferred in B	Not Inferred in B
**Present in A**	TP	FN
**Absent in A**	FP	TN

Confusion matrix. TP = true positive, FN = false negative, FP = false positive, TN = true negative.

The networks are represented in terms of their respective adjacency matrices *C*, such that each connection from gene *i *to gene *j *implies *C_ij _*= 1, with *C_ij _*= 0 otherwise. Then, in order to quantify the quality of the inferred network, the similarity measurements [60] widely used to compare inference methods were adopted, being calculated as follows:(7)

Since the measurements on Equation 7 are not independent of each other, it was adopted the geometrical average between the ratios of correct ones *PPV *(Positive Predictive Value, also known as accuracy or precision) and correct zeros (*Specificity*), observing the ground-truth matrix *A *and the inferred matrix *B*. In this way, both coincidences and differences are taken into account by these measures, thus implying the maximum similarity to be obtained for values near 1.

## Authors' contributions

FML wrote the software code, FML and EAO analyzed the data and wrote the manuscript. RMCJ participated in the design and coordination of the study. All authors contributed to, read and approved the final manuscript.

## References

[B1] Bansal M Belcastro V Ambesi-Impiombato A di Bernardo D How to infer gene networks from expression profiles Mol Syst Biol 2007 3 78 17299415 10.1038/msb4100120 PMC1828749

[B2] Hovatta I Kimppa K Lehmussola A Pasanen T Saarela J Saarikko I Saharinen J Tiikkainen P Toivanen T Tolvanen M DNA microarray data analysis 2005 2 CSC, Scientific Computing Ltd

[B3] DREAM DREAM: Dialogue for Reverse Engineering Assessments and Methods Gustavo Stolovitzky and Andrea Califano and Robert Prill and Julio Saez Rodriguez 2009 http://wiki.c2b2.columbia.edu/dream/

[B4] Husmeier D Sensitivity and specificity of inferring genetic regulatory interactions from microarray experiments with dynamic Bayesian networks Bioinformatics 2003 19 17 2271 2282 10.1093/bioinformatics/btg313 14630656

[B5] Tsallis C Possible generalization of Boltzmann-Gibbs statistics Journal of Statistical Physics 1988 52 479 487 10.1007/BF01016429

[B6] Abe S Tsallis entropy: how unique? Continuum Mechanics and Thermodynamics 2004 16 3 237 244 10.1007/s00161-003-0153-1

[B7] Cheng J Bell DA Liu W Learning belief networks from data: an information theory based approach CIKM '97: Proceedings of the sixth international conference on Information and knowledge management 1997 New York, NY, USA: ACM 325 331

[B8] Liang S Fuhrman S Somogyi R Reveal: a general reverse engineering algorithm for inference of genetic network architectures Proceedings of the Pacific Symposium on Biocomputing 1998 18 29 http://psb.stanford.edu/psb-online/proceedings/psb98/abstracts/p18.html 9697168

[B9] Friedman N Inferring Cellular Networks Using Probabilistic Graphical Models Science 2004 303 5659 799 805 10.1126/science.1094068 14764868

[B10] Margolin A Basso KN Wiggins C Stolovitzky G Favera R Califano A ARACNE: An algorithm for the reconstruction of gene regulatory networks in a mammalian cellular context BMC Bioinformatics 2006 7 Suppl 1 S7 10.1186/1471-2105-7-S1-S7 16723010 PMC1810318

[B11] Barrera J Cesar RM Jr Martins DC Jr Vencio RZN Merino EF Yamamoto MM Leonardi FG Pereira CAB Portillo HA McConnell P, Lin SM, Hurban P Constructing probabilistic genetic networks of *Plasmodium falciparum*, from dynamical expression signals of the intraerythrocytic development cycle Methods of Microarray Data Analysis V 2007 Springer US 11 26 http://dx.doi.org/10.1007/978-0-387-34569-7_2

[B12] Soranzo N Bianconi G Altafini C Comparing association network algorithms for reverse engineering of large-scale gene regulatory networks Bioinformatics 2007 23 13 1640 1647 10.1093/bioinformatics/btm163 17485431

[B13] Meyer PE Kontos K Lafitte F Bontempi G Information-theoretic inference of large transcriptional regulatory networks EURASIP Journal on Bioinformatics and Systems Biology 2007 2007 1 9 10.1155/2007/79879 PMC3171353 18354736

[B14] Zhao W Serpedin E Dougherty ER Inferring Connectivity of Genetic Regulatory Networks Using Information-Theoretic Criteria IEEE/ACM TCBB 2008 5 2 262 274 18451435 10.1109/TCBB.2007.1067

[B15] Dougherty J Tabus I Astola J Inference of gene regulatory networks based on a universal minimum description length EURASIP Journal on Bioinformatics and Systems Biology 2008 2008 1 11 http://dx.doi.org/10.1155/2008/482090 10.1155/2008/482090 PMC3171396 18437238

[B16] Lopes FM Martins DC Jr Cesar RM Jr Comparative study of GRNs inference methods based on feature selection by mutual information 2009 IEEE International Workshop on Genomic Signal Processing and Statistics (GENSIPS 2009), IEEE Signal Proc Soc, 345 E 47TH ST, New York, NY 10017 USA: IEEE 2009 110 113 http://dx.doi.org/10.1109/GENSIPS.2009.5174334 [7th IEEE International Workshop on Genomic Signal Processing and Statistics (GENSIPS 2009), Minneapolis, United States, MAY 17-19, 2009]

[B17] Kaleta C Gohler A Schuster S Jahreis K Guthke R Nikolajewa S Integrative inference of gene-regulatory networks in Escherichia coli using information theoretic concepts and sequence analysis BMC Systems Biology 2010 4 116 10.1186/1752-0509-4-116 20718955 PMC2936295

[B18] Chaitankar V Ghosh P Perkins E Gong P Deng Y Zhang C A novel gene network inference algorithm using predictive minimum description length approach BMC Systems Biology 2010 4 Suppl 1 S7 10.1186/1752-0509-4-S1-S7 20522257 PMC2880413

[B19] Kim DC Wang X Yang CR Gao J Learning biological network using mutual information and conditional independence BMC Bioinformatics 2010 11 Suppl 3 S9 10.1186/1471-2105-11-S3-S9 20438656 PMC2863068

[B20] Altay G Emmert-Streib F Inferring the conservative causal core of gene regulatory networks BMC Systems Biology 2010 4 132 10.1186/1752-0509-4-132 20920161 PMC2955605

[B21] Lopes FM Cesar RM Jr Costa LdF AGN Simulation and Validation Model Advances in Bioinformatics and Computational Biology, Proceedings, Volume 5167 of Lecture Notes in Bioinformatics, Springer-Verlag Berlin 2008 169 173 http://dx.doi.org/10.1007/978-3-540-85557-6_17

[B22] Lopes FM Cesar RM Jr Costa LdF Gene expression complex networks: synthesis, identification and analysis Journal of Computational Biology 2011 15 1 15 cmb.2010.0118 10.1089/cmb.2010.0118 21548810

[B23] Newman MEJ The Structure and Function of Complex Networks SIAM Review 2003 45 2 167 256 10.1137/S003614450342480

[B24] Boccaletti S Latora V Moreno Y Chavez M Hwang DU Complex networks: Structure and dynamics Physics Reports 2006 424 4-5 175 308 10.1016/j.physrep.2005.10.009

[B25] Costa LdF Rodrigues FA Travieso G Villas-Boas PR Characterization of complex networks: a survey of measurements Advances in Physics 2007 56 167 242 10.1080/00018730601170527

[B26] Lopes FM Martins DC Jr Cesar RM Jr Feature selection environment for genomic applications BMC Bioinformatics 2008 9 451 10.1186/1471-2105-9-451 18945362 PMC2655091

[B27] Lopes FM de Oliveira EA Cesar RM Jr Analysis of the GRNs inference by using Tsallis entropy and a feature selection approach Progress in Pattern Recognition, Image Analysis, Computer Vision, and Applications, Proceedings, Volume 5856 of Lecture Notes in Computer Science, Springer-Verlag Berlin 2009 473 480 http://dx.doi.org/10.1007/978-3-642-10268-4_55 [14th Iberoamerican Congress on Pattern Recognition (CIARP 2009), Guadalajara, Mexico, NOV 15-18, 2009]

[B28] Gray RM Entropy and Information Theory 1990 1 Springer-Verlag

[B29] Clausius R The mechanical theory of heat 1879 London: Macmillan

[B30] Boltzmann L Uber die Beziehung eines allgemeine mechanischen Satzes zum zweiten Haupsatze der Warmetheorie Math-Naturwissenschaften 1877 75 67 73

[B31] Tsallis C Gell-Mann M Sato Y Extensivity and entropy production Europhysics News 2005 36 6 186 189 10.1051/epn:2005602

[B32] Fermi E Thermodynamics 1956 New York: Dover Publications

[B33] Tsallis C What should a statistical mechanics satisfy to reflect nature? Physica D: Nonlinear Phenomena 2004 193 1-4 3 34 10.1016/j.physd.2004.01.006

[B34] Shannon CE A mathematical theory of communication Bell System Technical Journal 1948 27 379 423 623-656

[B35] Khinchin AI Mathematical Foundations of Information Theory. Dover 1957

[B36] Kullback S Information Theory and Statistics 1959 Wiley

[B37] dos Santos RJV Generalization of Shannon's theorem for Tsallis entropy Journal of Mathematical Physics 1997 38 8 4104 4107 10.1063/1.532107

[B38] Abe S Axioms and uniqueness theorem for Tsallis entropy Physics Letters A 2000 271 1-2 74 79 10.1016/S0375-9601(00)00337-6

[B39] Furuichi S Information theoretical properties of Tsallis entropies Journal of Mathematical Physics 2006 47 2 023302 10.1063/1.2165744

[B40] Wilk G Wlodarczyk Z Example of a possible interpretation of Tsallis entropy Physica A: Statistical Mechanics and its Applications 2008 387 19-20 4809 4813 10.1016/j.physa.2008.04.022

[B41] Borland L Plastino AR Tsallis C Information gain within nonextensive thermostatistics Journal of Mathematical Physics 1998 39 12 6490 6501 10.1063/1.532660

[B42] Tsallis C Generalized entropy-based criterion for consistent testing Phys Rev E 1998 58 2 1442 1445 10.1103/PhysRevE.58.1442

[B43] Kauffman SA Metabolic stability and epigenesis in randomly constructed genetic nets Journal of Theoretical Biology 1969 22 3 437 467 10.1016/0022-5193(69)90015-0 5803332

[B44] Kauffman SA The origins of order: Self-organization and selection in evolution 1993 Oxford University Press

[B45] Erdös P Rényi A On random graphs Publ Math Debrecen 1959 6 290 297

[B46] Barabási AL Albert R Emergence of scaling in random networks Science 1999 286 5439 509 512 10.1126/science.286.5439.509 10521342

[B47] Stuart JM Segal E Koller D Kim SK A gene-coexpression network for global discovery of conserved genetic modules Science 2003 302 5643 249 255 10.1126/science.1087447 12934013

[B48] Albert R Scale-free networks in cell biology J Cell Sci 2005 118 21 4947 4957 10.1242/jcs.02714 16254242

[B49] Marbach D Schaffter T Mattiussi C Floreano D Generating Realistic In Silico Gene Networks for Performance Assessment of Reverse Engineering Methods Journal of Computational Biology 2009 16 2 229 239 10.1089/cmb.2008.09TT 19183003

[B50] Pudil P Novovičová J Kittler J Floating Search Methods in Feature-Selection Pattern Recognition Letters 1994 15 11 1119 1125 10.1016/0167-8655(94)90127-9

[B51] Martins DC Jr Cesar RM Jr Barrera J W-operator window design by minimization of mean conditional entropy Pattern Analysis & Applications 2006 9 139 153 10.1007/s10044-006-0031-021547947

[B52] Shmulevich I Dougherty ER Genomic Signal Processing 2007 New Jersey: Princeton University Press

[B53] Kauffman S Peterson C Samuelsson B Troein C Random Boolean network models and the yeast transcriptional network PNAS 2003 100 25 14796 14799 10.1073/pnas.2036429100 14657375 PMC299805

[B54] Serra R Villani M Semeria A Genetic network models and statistical properties of gene expression data in knock-out experiments Journal of Theoretical Biology 2004 227 149 157 10.1016/j.jtbi.2003.10.018 14969713

[B55] Shmulevich I Kauffman SA Aldana M Eukaryotic cells are dynamically ordered or critical but not chaotic PNAS 2005 102 38 13439 13444 10.1073/pnas.0506771102 16155121 PMC1224670

[B56] Li S Assmann SM Albert R Predicting Essential Components of Signal Transduction Networks: A Dynamic Model of Guard Cell Abscisic Acid Signaling PLoS Biol 2006 4 10 e312 10.1371/journal.pbio.0040312 16968132 PMC1564158

[B57] Davidich MI Bornholdt S Boolean Network Model Predicts Cell Cycle Sequence of Fission Yeast PLoS ONE 2008 3 2 e1672 10.1371/journal.pone.0001672 18301750 PMC2243020

[B58] Shmulevich I Dougherty ER Kim S Zhang W Probabilistic Boolean Networks: a rule-based uncertainty model for gene regulatory networks Bioinformatics 2002 18 2 261 274 10.1093/bioinformatics/18.2.261 11847074

[B59] Webb AR Statistical Pattern Recognition 2002 2 John Willey & Sons

[B60] Dougherty ER Validation of Inference Procedures for Gene Regulatory Networks Current Genomics 2007 8 6 351 359 10.2174/138920207783406505 19412435 PMC2671720

